# Tailoring Mechanically Robust Poly(*m*-phenylene isophthalamide) Nanofiber/nets for Ultrathin High-Efficiency Air Filter

**DOI:** 10.1038/srep40550

**Published:** 2017-01-11

**Authors:** Shichao Zhang, Hui Liu, Xia Yin, Zhaoling Li, Jianyong Yu, Bin Ding

**Affiliations:** 1 State Key Laboratory for Modification of Chemical Fibers and Polymer Materials, College of Materials Science and Engineering, Donghua University, Shanghai 201620, China; 2 Nanofibers Research Center, Modern Textile Institute, Donghua University, Shanghai 200051, China; 3 Key Laboratory of Textile Science & Technology, Ministry of Education, College of Textiles, Donghua University, Shanghai 201620, China

## Abstract

Effective promotion of air filtration applications proposed for fibers requires their real nanoscale diameter, optimized pore structure, and high service strength; however, creating such filter medium has proved to be a tremendous challenge. This study first establishes a strategy to design and fabricate novel poly(*m*-phenylene isophthalamide) nanofiber/nets (PMIA NF/N) air filter via electrospinning/netting. Our strategy results in generation of a bimodal structure including a scaffold of nanofibers and abundant two-dimensional ultrathin (~20 nm) nanonets to synchronously construct PMIA filters by combining solution optimization, humidity regulation, and additive inspiration. Benefiting from the structural features including the true nanoscale diameter, small pore size, high porosity, and nets bonding contributed by the widely distributed nanonets, our PMIA NF/N filter exhibits the integrated properties of superlight weight (0.365 g m^−2^), ultrathin thickness (~0.5 μm), and high tensile strength (72.8 MPa) for effective air filtration, achieving the ultra-low penetration air filter level of 99.999% and low pressure drop of 92 Pa for 300–500 nm particles by sieving mechanism. The successful synthesis of PMIA NF/N would not only provide a promising medium for particle filtration, but also develop a versatile platform for exploring the application of nanonets in structural enhancement, separation and purification.

Atmospheric particulate matter (PM) pollution has recently become one of the most serious environmental issues, in particular, in China. Numerous studies have shown that exposure to PM_2.5_ (aerodynamic diameter ≤2.5 μm) leads to direct and indirect adverse effects on people’s health status and living, visibility, climate, and ecosystems^1^,^2^,^3^. Nowadays, the fibrous filters have been widely used for PM filtration due to their fascinating features, such as relatively energy-efficient, cost-effectiveness, ease of scalable fabrication, changeable filter design, and application universality^4^,^5^. In view of their micro-sized fiber diameter, the traditional filter media including melt-blown fibers, spun-bonded fibers, and glass fibers inevitably suffer from various application drawbacks, involving inadequate filtration efficiency, high air resistance and depth loading characteristics (particles would penetrate into the media, causing the limited regeneration and recycling ability), as well as they are incapable of capturing the ultrafine particles^6^,^7^,^8^. Several strategies based on the dramatic increase in the packing density and basis weight (for all fibrous materials), combined with electret treatment by charging technologies (usually for the melt-blown and spun-bonded fibers), have resulted in successful improvement in the filtration efficiency. However, the hugely increased fiber deposition amounts and easy-degraded electret effect, lead to the deficiency of the filters, like bulkiness, the compromise between air flow and filter efficiency, and safety hazards from charge dissipation^9^.

Recently, benefitting from the decrease of fiber diameter, nanofibrous filters have gained growing attention by virtue of their enhanced filtration performance in actual operating environments^10^,^11^. Compared to various methods including drawing, flash-spinning, sea-island spinning and template synthesis, electrospinning has been widely regarded as a robust and scalable nanotechnology for producing nanofibers with organic/inorganic components and designed structures^12^,^13^,^14^,^15^. Many electrospun fibers (diameter 100–1000 nm), including polyurethane (PU)^16^, polyacrylonitrile (PAN)^17^,^18^, polyvinyl chloride/PU^5^, polyamide-56^19^, poly(lactic acid)^20^, and nylon-66^21^, have been carefully fabricated and gained significantly improved filtration performance^22^. Nevertheless, some drawbacks still remain, such as limited structural controllability, weak mechanical properties, low production rates, and a low quality factor indicating an inadequate filtration performance. Considering the existing theory, further decrease in the fiber diameter to the real nanoscale (<100 nm) can substantially improve the material utilization and the resultant properties^23^,^24^. Several approaches involving liquid jet stretch enhancement^25^, core–shell or multicomponent spinning combined with subsequent component removal method^26^,^27^, and spinning of extremely diluted solution^28^, have been established to further decrease the diameter of nanofibers. However, there are inherent limits on the refining ability (the diameter of the existing nanofibers are usually >50 nm), low production capacity, and tedious process to present major challenges in the thinning of nanofibers which must be addressed before further improving their application performance in air filtration.

Electrospinning/netting (ESN), as an advanced electro-hydrodynamics technique in nanoscience and nanotechnology fields, draws significant attention by virtue of the capacity to the one-step preparation of two-dimensional (2D) “nanonets” with ultrathin diameter (~20 nm), stable pore size, and large quantities^29^,^30^. Benefiting from the true nanoscale diameter which can facilitate the “slip effect” of air molecules, and the small pore size which can completely sieve ultrafine particles, the resultant 2D nanonets stand out as a promising material for promotion of air filtration applications^21^,^31^. Previously, nylon-66 NF/N filters were first explored and prepared in our group. These filters showed a capture efficiency of 99.9% and a rather high pressure drop of ~200 Pa owing to their limited nanonet coverage rate and large thickness of 19.05 μm, which could not satisfy the demands for practical applications^21^. Subsequently, the polyamide-56 NF/N filter gained improved removal efficiency of >99.99%; however, its air resistance was rather high (>110 Pa) due to the bonding structures^19^. Recently, nylon-6 NF/N filters with cavity structures (enlarged voids between the adjacent fibers) were designed and fabricated by embedding PAN nanofibers or poly(*m*-phenylene isophthalamide) staple fibers into the membranes, and their air permeability was effectively enhanced. However, the nanonets obtained from nylon-6 system, in general, suffer from the low formation probability of nanonets, inhomogeneous pore size, and partial non-porous films. In order to achieve a high filtration efficiency (~99.99%), it is unavoidable that more nylon-6 fibers would need to be deposited to increase the nanonets content in the membrane. Therefore, the deficiency of the resulting filters is large basis weight (>1.1 g m^−2^) and high thickness (>20 μm), both of which would pose a large obstacle for reducing the air resistance of the filters^31^,^32^. Moreover, their structural stability still remained a great challenge due to the weak mechanical properties of the nanofibers and the inevitable agglomeration of the staple fibers. Despite many past efforts for promoting application capacity of air filters, we concluded that following three bottleneck problems still remain: (i) achievement of high efficiency with low air resistance, in particular, for ultra-low penetration air (ULPA) filter level and capturing airborne particles based on the safest sieving principle (a capture mechanism getting rid of the negative impacts from high airflow speed, electret failure and high humidity), (ii) resolution of the bulkiness and material consumption for the high performance-cost ratio, and (iii) maintenance of the essential mechanical properties, especially for the real nanofibers (<100 nm).

Herein, we designed and fabricated a novel NF/N membrane with characteristics including superlight weight, ultra-low thickness, and mechanically robust properties for ULPA filtration via the ESN technology. The structure and filtration performance of PMIA membranes made from 1D nanofibers were designed and optimized by regulating PMIA concentration in the precursor solutions. Moreover, novel PMIA NF/N membranes composed of the scaffold nanofibers and widely distributed 2D nanonets with true nanoscale diameter (~20 nm) were thoroughly optimized by tuning the relative humidity (RH) and the dodecyl trimethyl ammonium bromide (DTAB) concentration in PMIA solutions. Furthermore, the filtration performance, pore structures, and mechanical properties of the PMIA NF/N membranes were systematically investigated, and *in situ* scanning electron microscopy (SEM) observation and 3D model simulation for elaborating the filtration process and a three-step break mechanism were studied to revel the core role of the 2D nanonets in guaranteeing the practical service performance of PMIA NF/N filtration membrane.

## Results

### Construction of scaffold structures

**Figure 1:**
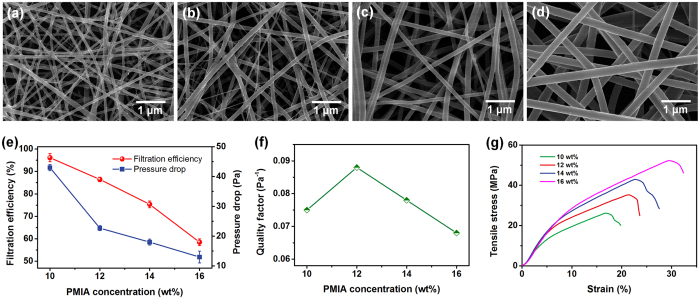
Morphology, filtration performance, and mechanical property of PMIA scaffold nanofiber filters. FE-SEM images of PMIA membranes obtained from solutions with PMIA concentration of (**a**) 10, (**b**) 12, (**c**) 14, and (**d**) 16 wt% at RH of 40%. (**e**) Filtration efficiency and pressure drop, (**f**) quality factor, and (**g**) stress-strain curves of PMIA nanofiber membranes formed from solutions with different PMIA concentrations at RH of 40%. The filtration performance in (**e**) and (**f**) is tested using PMIA membrane with basis weight of ~0.125 g m^−2^.

Optimization of the filter design to achieve excellent filtration capacity and durability for ultrafine particle filtration requires endowing the membranes with optimized scaffold structures and robust mechanical properties^32^. The field emission (FE)-SEM images of PMIA nanofibers obtained from solutions with different PMIA concentrations under RH of 40% are shown in Fig. 1a–d. The images exhibit a 3D structure composed of randomly deposited nanofibers in the form of nonwoven fabric, which could provide the desired tortuous channels for air transmission and particle intercept. Clearly, the nanofibers obtained using 10, 12, 14, and 16 wt% PMIA solutions exhibit an increasing average diameter of 125, 160, 185, and 288 nm, respectively, and the fibers tend to be much more uniform and smooth. This increasing fiber diameter could be attributed to the decreased conductivity and sharply increased viscosity of PMIA solutions (Table S1), which could significantly suppress the elongation and thinning of charged jets during spinning^33^. Furthermore, few randomly distributed nanowires with diameter of 30–50 nm appeared in the membranes obtained using solutions with lower PMIA concentrations (10 and 12 wt%), resulting in the membranes with bimodal diameters which were beneficial to the particle adhesion during the filtration process. This unexpected phenomenon was attributed to the increased conductivity of the solution that followed a higher charge distribution on the flying jets, thus leading to the generation of a stronger electric repulsion, resulting in the splitting up of the electrospun jets^30^.

Herein we employed 300–500 nm sodium chloride (NaCl) aerogel particles which were charge neutralized using an electrostatic neutralization device (NaCl particles captured the ions with opposite charges generated from air molecules, see the details in Supplementary Methods) to investigate the filtration performance of PMIA membranes^32^. The membranes (~0.125 g m^−2^) fabricated from 10, 12, 14, and 16 wt% PMIA solutions exhibited different filtration efficiencies of 96.145%, 86.452%, 75.367%, and 58.508%, while their pressure drops were 43, 22.7, 18, and 13 Pa, respectively, revealing a synchronous decrease with increasing PMIA concentration, as shown in Fig. 1e. Obviously, the increased fiber diameter allowed the pore size stacked by the fibers to increase sharply, favoring both the particles penetration and airflow through the membranes^18^. Moreover, the randomly distributed nanowires in membranes from 10 and 12 wt% PMIA solutions could slightly enhance the effective contact area for capturing NaCl particles, and thus endowed the membranes with higher removal efficiency. To effectively evaluate the integrated performance of air filters, the quality factor (*QF*) was introduced and calculated based on the formula in existing reports^8^,^34^: *QF* = −ln (1 − *η*)/Δ*p*, in which the *η* was removal efficiency and the Δ*p* was pressure drop. Figure 1f demonstrates that the *QF* values of PMIA nanofiber membranes fabricated using 10, 12, 14, and 16 wt% PMIA solutions are 0.075, 0.088, 0.078, and 0.068 Pa^−1^, respectively, further confirming the competitive balance between fiber diameter and packing density towards constructing the scaffold-like structure for air filtration, in particular, for energy conservation and emissions reduction fields. Furthermore, the PMIA nanofiber membrane obtained from 12 wt% PMIA solution exhibited outstanding mechanical properties, such as tensile strength of 35.3 MPa, Young’s modulus of 0.312 GPa, and toughness of 5.57 MJ m^−3^ (Figs 1g and S1), indicating the essential mechanical strength which is required to remain the structural integrity during their subsequent processing.

### 2D nanonet structure design

**Figure 2:**
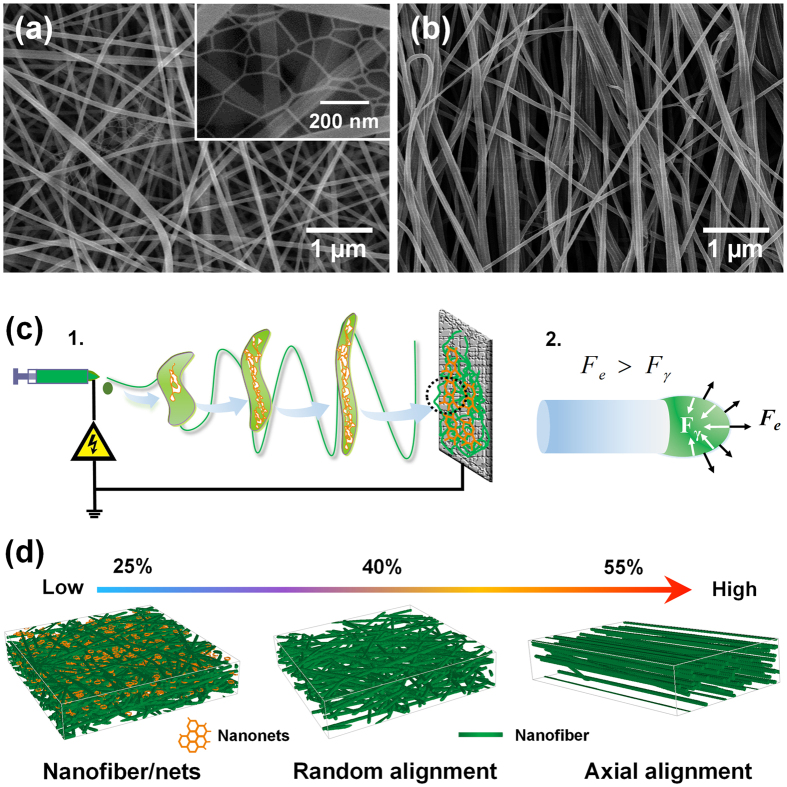
Structural design of PMIA nanofibrous aggregation. FE-SEM images of PMIA membranes obtained at various RH of (**a**) 25% and (**b**) 55%. (**c**) Schematic illustration of (1) the fabrication process of PMIA NF/N membranes deposited on the nonwoven substrate, and (2) the major forces acting on the Taylor cone. (**d**) Illustration of stacking structures of PMIA nanofibrous aggregates formed at various RH ranging from 25% and 40% to 55%.

During the ESN process (Fig. 2c), the RH acts as a vitally important operational parameter for regulating the morphology and structure of NF/Ns, because RH is closely related to the solvent evaporation and charge dissipation of the charged liquids^35^,^36^. Figure 2a and b show the FE-SEM images of PMIA membranes obtained at RH of 25% and 55%, respectively. Under RH of 25%, novel 2D fishnet-like structures (nanonets), comprising interlinked 1D ultrathin nanowires (~20 nm) and displaying geometric characteristic with topological Steiner networks (inset of Fig. 2a), appeared in the membrane. According to the numerical models proposed for elaborating the origin and evolution of poly(acrylic acid) (PAA) nanonets in our previous study^30^, it is clear that the charge to mass ratio (*e/m*) should exceed the critical value of “droplet mode” (Fig. 2c) and the appropriate phase separation is required to facilitate the evolution of Steiner-tree structures. Benefiting from the high conductivity caused by the ionic liquid lithium chloride/*N,N*-dimethylacetamide (LiCl/DMAc), it was easier to endow the Taylor cone with significantly more charges. Herein, we studied for the first time the charged situation, stress competition, and deformation of the charged liquid on Taylor cone apex for the PMIA/DMAc system (see the detailed derivation in Supplementary Information). After careful calculation by using the following equations based on the precondition between Coulomb repulsion *F*_*e*_ and hydrostatic pressure *F*_*γ*_ (*F*_*e*_ > *F*_*γ*_, Fig. 2c(2)):


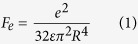



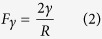



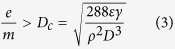


We obtained the theoretical threshold of *e/m* (droplet threshold *D*_*c*_, Equation 3) of the fluid for ESN using the solution properties and electric field parameters; and the value of *e/m* was 1.19 c kg^−1^, as listed in Table S2. Fortunately, the charged fluid from 12 wt% PMIA solution under RH of 25% possessed a much higher *e/m* of 1.33 c kg^−1^, indicating the formation capacity of the droplets, which is in well agreement with the SEM observation (Fig. 2a). Owing to various intriguing properties such as extremely small diameter and pore size (usually smaller than the most penetration particle size (MPPS) of 300 nm)^4^, large specific surface, and enhanced interconnectivity, the resulting 2D PMIA nanonets act as a promising candidate for air filtration application. With increasing the RH to 40%, the nanonets disappeared (Fig. 1b), which can be ascribed to lower *e/m* of 0.077 c kg^−1^ (Table S2) of the liquid on Taylor cone apex caused by the rapid charge dissipation in the humid environment, which failed to generate the original charged droplets for nanonets^29^,^31^. Further increasing the RH to 55% caused the PMIA membrane to exhibit an obvious aligned structure without nanonets (Fig. 2b), which can be explained based on the competition between the effects of phase separation and charge loss, and this result agreed well with our previous reports^33^,^37^. Therefore, the obvious conclusion could be drawn that careful optimization of the RH during ESN could yield varieties of PMIA membranes with designed structures (with or without nanonets and aligned or non-aligned), as illustrated in Fig. 2d, which could satisfy the requirement in various application fields, such as structural materials, filtration, separation, and so forth.

**Figure 3:**
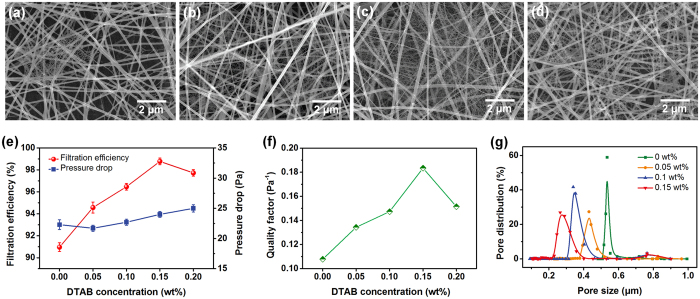
Morphology, filtration performance, and pore structure of PMIA nanofiber/nets filters. FE-SEM images of PMIA membranes prepared from solutions with different DTAB concentrations of (**a**) 0.05, (**b**) 0.1, (**c**) 0.15, and (**d**) 0.2 wt% at RH of 25%. (**e**) Filtration efficiency and pressure drop, (**f**) quality factor, and (**g**) pore size distribution of PMIA NF/N membranes (~0.125 g m^−2^) with different DTAB concentrations.

Following the accessibility to the novel PMIA nanonet structures, the further goal was to substantially improve the quantity of 2D nanonets, i.e., their coverage rates, in the membrane. In daily life, soap bubbles usually show Steiner-tree networks spontaneously, indicating the stable geometry structure similar to the highly interconnected nanonets. Inspired by this, we employed a surfactant, DTAB, to endow the PMIA solutions with the designed surface tension and conductivity to enhance the formation probability of the droplets and to optimize the pore structures of the nanonets^38^,^39^. The typical FE-SEM images of PMIA NF/N membranes fabricated using PMIA solutions with various DTAB contents are presented in Fig. 3a–d. Compared to the limited nanonet coverage rate (<10%) in pristine PMIA membrane (Fig. 2a), the NF/Ns membranes formed using solution with 0.05, 0.1, and 0.15 wt% DTAB showed sharply increased coverage rates of 25%, 58%, and 100%, respectively, even multi-level nanonets appeared in PMIA membranes with 0.15 wt% DTAB. This phenomenon could be attributed to the decreased surface tension and the increased conductivity (Table S1), both of which could enhance the *e/m* of the liquid on the Taylor cone apex. The *e/m* of PMIA solution with 0.15 wt% DTAB was 3.97 c kg^−1^, which was much higher than its corresponding droplet threshold of 1.05 c kg^−1^, as listed in Table S2. With the effective enhancement in charge capacity, the generation probability of charged droplets sharply increased owing to the enhanced instability of the Taylor cone apex. Moreover, the intermolecular hydrogen bonding among PMIA macromolecules may facilitate the formation of the Steiner-tree structure by virtue of their enhanced binding force during the evolution process of nanonets from “liquid film” to “pore structures”^30^. Therefore, combining the enhanced formation probability and stable phase separation of the droplets, the PMIA membrane containing completely distributed nanonets was successfully fabricated, as displayed in Fig. 3c. Moreover, benefiting from the more systematic and targeted regulation process, the PMIA nanonets effectively got rid of the defects which PAA and nylon-6 nanonets systems suffered from, such as the inhomogeneous nanowire diameter, pore size, and partial non-porous films, etc. (the representative FE-SEM images of PAA, nylon-6, and PMIA NF/Ns were shown in Figure S2). Furthermore, the PMIA nanonets system significantly enhanced the nanonet formation probability compared to other nanonets systems; i.e., it could achieve the completely distributed nanonets with much less fiber deposition amounts, indicating the tendency of synchronously achieving high removal efficiency and low air resistance. However, when 0.2 wt% DTAB was added into the PMIA solution, the resulting membrane exhibited a slightly deteriorated nanonets coverage of 92%. This result could be ascribed to the excessive increase of conductivity of PMIA solution, as demonstrated in Table S1. Thus, significantly increased drafting force originating from more residual charges on the droplets led to the breaking of the partial nanonets during the flight of the phase separated liquid film, and this resulted in larger pore size of the nanonets than those of membranes obtained from solutions with lower DTAB concentrations^21^,^38^.

Plotting the filtration efficiencies, pressure drops, and *QF* values of PMIA NF/N membranes with various nanonet coverage rates versus different DTAB concentrations, respectively, revealed that 2D nanonets significantly influenced the removal performance of PMIA membranes for air filtration. Fig. 3e exhibits that the filtration efficiencies of PMIA membranes (~0.125 g m^−2^) obtained from solutions with 0, 0.05, 0.1, 0.15, and 0.2 wt% DTAB are 90.964%, 94.753%, 96.466%, 98.775%, and 97.729%, while their relevant pressure drops are 22.3, 21.7, 22.7, 24, and 25 Pa, respectively, indicating a significant increasing trend (0–0.15 wt% DTAB) and then slightly deteriorated for the removal efficiency, together with a virtually unchanged air resistance. This result completely proved the novel nanonets to be an ideal filtration medium that the scientist craved to obtain for the practical applications. The PMIA NF/N membrane formed from solution with 0.15 wt% DTAB was endowed with the highest *QF* value of 0.183 Pa^−1^, further and intuitively revealing the crucial role of nanonets in enhancing their filtration performance. Moreover, the *QF* variation trend was consistent with the change of the nanonet coverage rate obtained by the FE-SEM analysis. To further reveal the unique superiority of the 2D nanonets function as air filter, we measured the pore size and distribution of PMIA NF/N membranes using a capillary flow porometer, as shown in Fig. 3g. Obviously, all PMIA membranes exhibit uniform and centralized pore distribution of 0.15–0.9 μm. Moreover, the cumulative pore size distribution for the membranes indicated that almost all the pores were concentrated at 0.28–0.55 μm, as shown in Figure S3. The enhanced nanonet structures through increasing DTAB content (0–0.15 wt%) endowed the PMIA membranes with reduced mean pore sizes ranging from ~0.56, ~0.44, ~0.37, to ~0.30 μm, as tabulated in Table S3. This change of the pore size could be ascribed to the enhanced nanonet content which provided abundant complanate pores with sizes of 200–300 nm, further confirming the significant contribution of the nanonets towards optimization of the pore structures of PMIA membrane. For the membrane with high porosity (Table S3), the airflow can have more path choices to pass through the medium; thus according to the minimal resistance principle, the length and tortuosity of the air penetration path can be significantly reduced, resulting in a lower air resistance which is in good agreement with the aforementioned filtration performance of PMIA NF/N filters. A more exciting conclusion could be drawn that the optimized PMIA NF/N membrane exhibited a mean flow pore size of 296 nm (smaller than the MPPS of 300 nm) and surface area of 26.89 m^2^ g^−1^ (Tables S3 and S4), indicating the complete physical sieving ability towards ultrafine airborne particles.

### Filtration progress simulation

**Figure 4:**
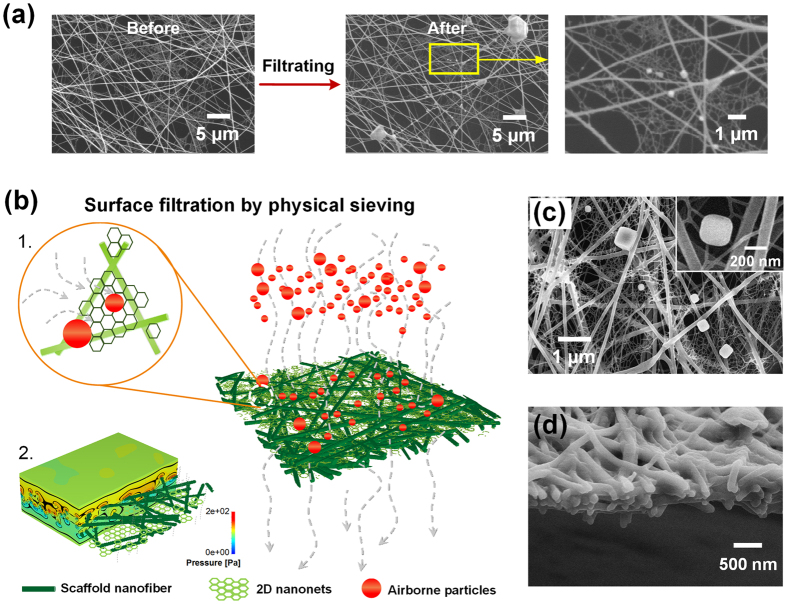
*In situ* time evolution study, 3D modeling, and morphology change of airborne particle capture by PMIA nanofiber/nets filters. (**a**) *In situ* study of NaCl aerosol particle capture by PMIA NF/N (~0.1 g m^−2^) characterized by SEM showing morphologies before and after the filtration process. (**b**) 3D model illustrating the filtration process of PMIA NF/N membrane for 300–500 nm particles by physical sieving and surface filtration: (1) absolute removal manner and (2) robust air permeability. FE-SEM images of (**c**) the top surface and (**d**) cross section of PMIA NF/N membrane with a basis weight of 0.365 g m^−2^ after filtration (air flow of 32 L min^−1^, testing for just 1 min).

The airborne particle removal process was *in situ* studied by SEM using a PMIA NF/N filter with partially distributed nanonets. Figure 4a shows the filter before and after capturing the particles. Obviously, the particles deposited almost in the region with nanonet structures, especially for the ultrafine ones. The PMIA nanonets could completely maintain their structural integrity during the filtration process. To elucidate the filtration process for our novel NF/Ns filter, a hypothetical 3D structure model was created by using FiberGeo procedure based on the structural information obtained from the FE-SEM observations and pore structure measurements^16^,^40^,^41^,^42^,^43^, as shown in Fig. 4b. Among the five proposed filtration mechanisms (interception, electrostatic attraction, inertial separation, diffusion, and sieving) for airborne particles, the sieving mechanism is the safest strategy that is desirable to be introduced into air filter designs due to its complete physical interception; however the other four mechanisms are based on the adsorption effect and are negatively impacted by external factors, such as humid environment, high wind speed, large ventilation volume, long-term service, and so on^44^. Based on the introduction of widely distributed nanonets with moderate and uniform pore size (200–300 nm), the PMIA membranes can even trap the MPPS particles by physical sieving (Fig. 4b(1)), which is unimaginable for the conventional filtration media. Moreover, benefiting from the 2D pore structure rather than the tortuous pore structure constructed by densely packing substantial amounts of fibers, significantly more open and unobstructed channels are generated in the PMIA membrane, which enhance the air permeability of the medium, thus resulting in much lower pressure drop (Fig. 4b(2)). Furthermore, the particle filtration by conventional fiber membranes in practical applications is primarily performed by deep bed filtration, which leads to a serious deterioration of their service life. Figure 4c demonstrates that almost all the NaCl particles can be captured on the surface of PMIA NF/N membranes rather than penetrating into the interior of the membranes, which belongs to the typical “surface filtration”. This filtration mechanism facilitates the cleaning of our PMIA NF/N filter through air back blowing and/or mechanical shaking, and thus can greatly prolong the life of the filters^21^. Moreover, in the case of the same/better filtration performance, PMIA NF/N filter possesses much smaller fiber deposition amounts than other electrospun membranes, let alone the conventional filtration media, which obviously reduces the thickness (~500 nm) of filter membrane and makes it much more lightweight (0.365 g m^−2^), as demonstrated in Fig. 4d and Table S5.

### Mechanical properties analysis

**Figure 5:**
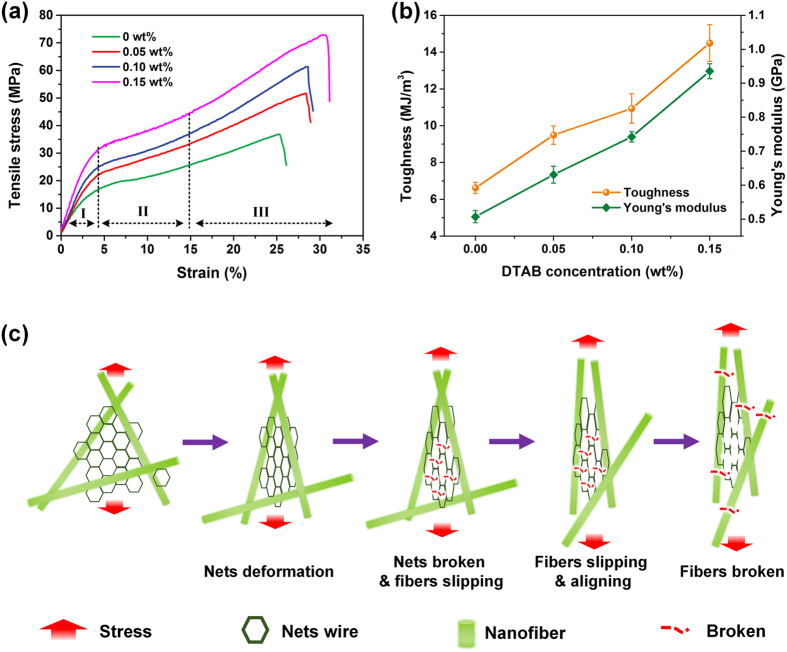
Analysis of mechanical properties of PMIA nanofiber/nets filters. (**a**) Tensile stress–strain curves and (**b**) toughness and Young’s modulus of PMIA NF/N membranes prepared from PMIA solutions with different DTAB concentrations at RH of 25%. (**c**) Illustration of three-step break mechanism of fracture process upon tensile stress.

The current strengthening strategies for the mechanical properties of certain electrospun fiber membranes, in general, include fiber alignment and fiber bonding; however, both of these approaches damage the filtration capacity of the membranes: the former deteriorates the capability to capture particles, while the latter always causes increased airflow resistance^5^,^33^. Hearteningly, our novel “nets bonding” strategy based on nanonet structures successfully enhanced the mechanical properties without compromising the filtration performance of PMIA NF/N membrane. Figure 5a and S4 show that the PMIA NF/N membranes fabricated with improved DTAB content at RH of 25% possess a tensile strength of 36.8, 51.6, 61.4, and 72.8 MPa, respectively, indicating a significant enhancement in contrast to the PMIA membranes without nanonets (35.3 MPa). Moreover, these PMIA NF/N membranes with widely distributed nanonets exhibited dramatically enhanced tensile strength compared to previously reported nanonets-based filter media, such as PA-56^19^, PA-6/PAN^31^, PA-6/PMIA staple fibers^32^, and so on (Table S6). As expected, compared to the relatively low Young’s modulus and toughness (Figure S1) of the pristine PMIA nanofibers, the membranes formed with increased DTAB concentration (0–0.15 wt%), i.e., with optimized coverage rate of nanonets, showed the enhanced toughness of 6.62, 9.49, 10.93, and 14.49 MJ m^−3^ and Young’s modulus of 0.507, 0.631, 0.742, and 0.935 GPa, respectively. The robust Young’s modulus could be ascribed to the dramatically reduced diameter of the nanowire in nanonets, because smaller fibers have fewer structural imperfections (such as the unavoidable chain ends, etc.), and a higher degree of molecular chain orientation along the fiber axis. Moreover, the highly interconnected nanonets resulted in an effective “net bonding” effect by virtue of the abundant bonding points between the nanowires and scaffold nanofibers. Consequently, the excellent mechanical properties of PMIA NF/N membrane, which were of vital importance in the post-processing of filter media, would accelerate its commercialization function as high efficiency particulate air (HEPA) filter and ULPA filter.

Further careful comparison of the stress–strain curve shape of PMIA NF/N membranes (Fig. 5a) and that of PMIA nanofiber membranes (Fig. 1g) indicated that the former presented a much more obvious gradient change with a larger slope, which matched very well with the mechanical properties mentioned above. It was clearly demonstrated that the PMIA membranes containing nanonet structures exhibited a linear elastic behavior at the initial stage under relatively low stress before reaching the yield point, and then the curves presented a nonlinear elastic behavior until the breakage occurred from the slip and “pull out” process of the scaffold nanofibers along the stress direction. This phenomenon can be elaborately clarified by a three-step break mechanism, as illustrated in Fig. 5c. When exposed to the external stress, the net structures tend to produce dynamic deformation from the “mesh” to “slit”, resulting in the rapidly increased and linear elastic behavior in the stage I. With continually increasing tensile stress (in the stage II), the nanonets show the tendency to break up and lose the reinforcement effect for the membrane, thus entire external load is shared through slipping and aligning of the scaffold fibers, corresponding to the relatively slow improvement of the tensile stress. Based on the further fiber slipping, certain aligned nanofiber membrane exhibits plastic deformation, which can be ascribed to the significant “pull out” of individual nanofibers (stage III). Finally, the PMIA NF/N membrane breaks due to the cumulative results of single nanofiber breakage.

### Filtration performance evaluation

**Figure 6:**
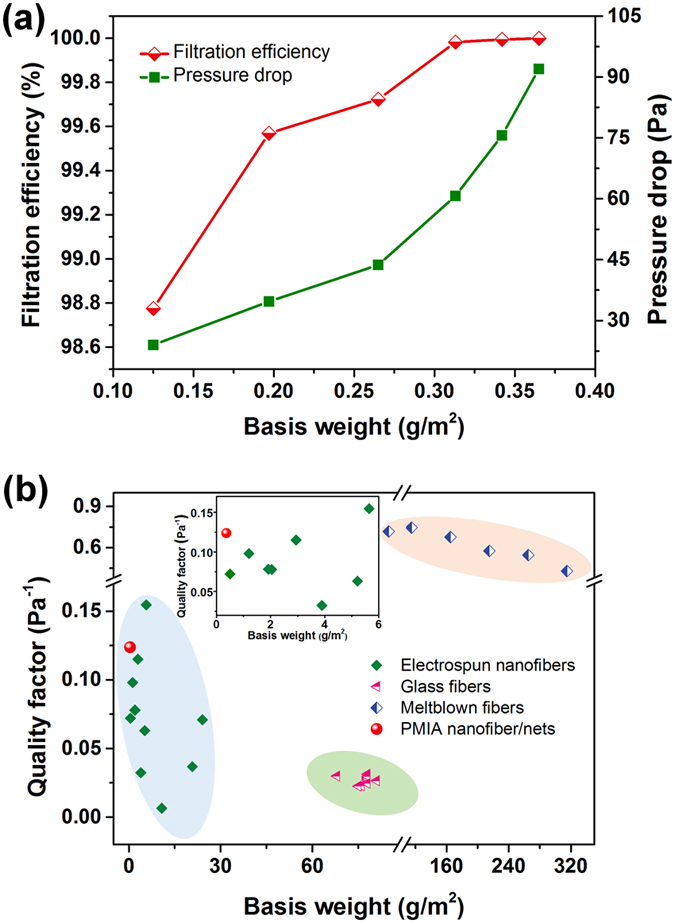
Evaluation of filtration performance of PMIA nanofiber/nets filters. (**a**) Filtration efficiency and pressure drop of PMIA NF/N filters with various basis weights. (**b**) Comparison of quality factor and basis weight between current filtration materials and PMIA NF/N membranes (all filtration media are tested at 32 L min^−1^). Inset shows the basis weight region of 0–6 g m^−2^, each symbol represents a type of polymer or a typical commercial product.

Figure 6a shows the filtration efficiency and pressure drop of PMIA NF/N filter with different basis weights under airflow of 32 L min^−1^. The filtration efficiencies of PMIA NF/N filtration media versus their increased basis weights are 98.775%, 99.569%, 99.723%, 99.982%, 99.994%, and 99.999%; while the relative pressure drops are 24, 34.7, 43.7, 60.7, 75.6, and 92 Pa, respectively. Noticeable conclusions could be drawn that, the removal efficiency improved sharply and then underwent gradual increase after the basis weight exceeded 0.313 g m^−2^, facilely achieving the HEPA filter level (>99.97%). Somewhat surprisingly, the optimized PMIA NF/N filter with a superlight weight of 0.365 g m^−2^ and ultrathin thickness of ~0.5 μm, exhibited an extremely high filtration efficiency of 99.999% (ULPA filter standard) for the ultrafine airborne particles while maintaining a low pressure drop of 92 Pa, indicating the key role of the novel nanonets in improving the application performance of the air filters. To further evaluate the time dependent behaviour (service life), we employed the testing method mentioned in our previous studies (as described in the Supplementary Method)^18^, to systematically investigate the dust holding capacity of the PMIA NF/N filters. Figure S5 exhibits that during the continuous silica particles feed, the dust holding capacities and pressure drops of the filter increase simultaneously, and achieve a rather high dust holding capacity of ~45 g m^−2^, thus confirming its longer service life benefited from the surface filtration and physical sieving manner. Furthermore, to study the efficacy of PMIA NF/N filters in a polluted air environment during hazy days, we performed a long-term recycling test for removing smoke PM_2.5_ from >500 μg m^−3^ (severely polluted level) to <35 μg m^−3^ (excellent level) in an artificial environment built by burning incense smokes. And, we named this capture process PM_2.5_ purification. Figure S6 demonstrates that PMIA NF/N filter used in this study could complete an air purification process in just ~13 min by virtue of its high removal efficiency and low air resistance. Moreover, the PM_2.5_ purification speed of this PMIA NF/N filter exhibited almost no change after 30 purification process cycles, indicating a robust removal capacity for the real PM in air.

To visually and comprehensively elaborate the filtration capacity and applications perspective of PMIA NF/N filter, a comparison of the *QF* and basis weight between the best filtration materials available around the world and PMIA NF/N medium was proposed^45^, as demonstrated in Fig. 6b and Table S5. To date, three major HEPA filter media including electret melt-blown fibers, superfine glass fibers, and nanofibers (in particular, electrospun nanofibers) have received world-wide attention in academic and/or industrial fields. Although the melt-blown fiber material with macro-sized diameter can achieve a rather high *QF* level by virtue of the electret effect and the unlimited increase of basis weight (>100 g m^−2^, even nearly 300 g m^−2^), it still suffers from the potential safety hazard due to electret degradation when exposed to chemicals and aerosols with opposite charges or high humidity environments, which is emphatically considered in the latest European standard (EN779: 2012)^46^. The glass medium works well at filtrating the fine particles with a slightly reduced basis weight and without electrostatic dependence, based on effective reduction of the fiber diameters (500–700 nm); however, the resulting extremely high air flow resistance leads to its relatively low *QF* level (<0.05 Pa^−1^). Electrospun nanofibers, as a promising media standing at the edge of novel air filters with high efficiency and low air resistance, have significantly aroused researchers’ interest because of their small diameters (100–700 nm)^15^. Figure 6b shows that electrospun nanofibers possess a relatively low basis weight (<20 g m^−2^, one third of that of glass media), and a comparable *QF* value (~0.06 Pa^−1^), indicating tremendous potential in air filtration based on the enhanced physical intercept effect. Among them, our novel PMIA NF/N filter stands out from all other HEPA filters by virtue of its superlight weight of 0.365 g m^−2^, even one fiftieth of that of conventional electrospun nanofiber media, two orders of magnitude lower than that of melt-blown fibers media; and an extremely high *QF* value of 0.12 Pa^−1^ as an ULPA filter.

## Discussion

The successful synthesis of nanonets based air filter develops a versatile platform for exploring the applications of nanonets in structural enhancement, separation, and purification. In this study, the PMIA NF/N filter was designed and fabricated as a model system for proof-of-concept; by considering the systematic formation models and regulation methods proposed as mentioned above, this discovery would pave the way for new types of nanonets based materials for various applications. For example, the biocompatible nanonets membranes (such as poly(lactic acid) and polycaprolactone) can be prepared to meet the requirements of tissue engineering. Moreover, some precursor polymers for carbon nanofibers (for e.g., PAN) can also be used to fabricate the nanonets and transform into carbon nanonets after the designed calcination process.

In conclusion, we designed and fabricated novel PMIA NF/N membrane, composed of the scaffold nanofibers and 2D ultrathin (~20 nm) nanonets for ULPA filtration via novel ESN technology. The PMIA NF/N membrane filter was systematically designed via constructing scaffold nanofibers that were realized by facilely regulating the PMIA concentration, and optimizing the 2D nanonets formation which was realized by regulating the RH and concentration of added DTAB, respectively. With the intriguing properties of superlight weight of 0.365 g m^−2^, ultrathin thickness of ~500 nm, and mechanical robustness of 72.8 MPa, the resulting PMIA NF/N filter possessed a high removal efficiency of 99.999% and a low air resistance of 92 Pa for 300–500 nm NaCl particles based on the synergistic effect of sieving manner and surface filtration. We believe that PMIA NF/N membrane used in this study can be used as a stand-alone filter or incorporated with window screening, respirator, HEPA filter and engine intake to achieve significantly clean living environment.

## Methods

### Materials

PMIA initial fibers (Conex^®^, 20 μm × 1 mm) were bought from TEIJIN Co., Ltd., Japan. *N,N*-dimethylacetamide (>98%) (DMAc) and silica nanoparticles (4–70 nm) were supplied by Shanghai Chemical Reagents Co., Ltd., China. Anhydrous lithium chloride (>99%) (LiCl) and DTAB (>99%) were obtained from Shanghai Aladdin Chemical Co., Ltd., China. All chemicals were of analytical grade and used as received without further purification.

### Preparation of PMIA nanofibrous membranes

PMIA precursor solution was obtained by dissolving PMIA initial fibers in the LiCl/DMAc solvent by magnetic stirring at 80 °C. The PMIA concentrations in the solutions were adjusted to 10, 12, 14, and 16 wt%, while the LiCl concentration was maintained at 2.5 wt%. PMIA/DTAB blended solutions were prepared by dissolving 12 wt% PMIA into the homogeneous LiCl/DMAc ionic liquid containing 0.05, 0.1, 0.15, and 0.2 wt% DTAB, respectively. Table S1 lists the detailed compositions, concentrations, and properties of the solutions mentioned above in the Supplementary Information.

All PMIA nanofibrous membranes were fabricated by using a DXES-3 ESN machine (SOF Nanotechnology Co., Ltd., China), as illustrated in Fig. 2c(1). The precursor solutions were transferred into a springe pump and squeezed at controllable rate of 0.1 mL h^−1^. The power supply of 30 kV was applied to the 6-G metal needles to facilitate the formation of liquid jets and charged droplets. The resultant PMIA membranes were deposited on the nonwoven substrate-covered grounded stainless roller (rotating rate: 80 rpm, spinneret-collector distance: 20 cm). The ambient temperature during ESN was maintained at 25 °C, while the RH was adjusted to 25%, 40%, and 55%, respectively. All the samples were vacuum-dried at 80 °C for 2 h to remove the residual charges and solvent before further use.

### Characterization

The properties of PMIA precursor solutions were systematically tested by the methods mentioned in our previous study^17^,^18^; however, the charge to mass ratio (*e/m*) of the charged fluid (jets and/or droplets) during ESN was measured by the typical “mesh target” method using a digital multi-meter (Fluke F15B+, Fluke electronic instrument Co., Ltd., China)^30^. Morphology of relevant samples was examined by using an S-4800 field emission scanning electron microscopy (FE-SEM) (Hitachi Ltd., Japan). The nanonet coverage rate was calculated by the “grid method” based on a series of SEM images; while the membrane thickness was tested using a CHY-C2 thickness gauge with precision of 0.1 μm (Labthink Co., Ltd., China). The CFP-1100AI capillary flow porometer (Porous Materials Inc., USA) was employed to characterize the pore structures of the relevant membranes. The tensile stress–strain curves were obtained by using an XQ-1C tensile tester (Shanghai New Fiber Instrument Co., Ltd., China) with a cross-head speed of 5 mm min^−1^. The size of the membrane specimen was 3 × 20 mm and at least 10 specimens from each membrane were tested for tensile behavior. Then, the Young’s modulus could be obtained by calculating the derivative of stress with respect to strain at low strain, i.e., the initial slope of the stress-strain curves. The filtration performance of PMIA membranes was tested by using an LZC-H filter tester (Huada Filter Technology Co., Ltd., China). Further, the detailed operation for measuring the *e/m* of the flying liquid during ESN and evaluation of the filtration performance (removal efficiency and pressure drop for filtrating NaCl particles, dust holding capacity for silica nanoparticles, and PM_2.5_ purification capacity for smoke particles) are provided in the Supplementary Information.

## Additional Information

**How to cite this article**: Zhang, S. *et al*. Tailoring Mechanically Robust Poly(*m*-phenylene isophthalamide) Nanofiber/nets for Ultrathin High-Efficiency Air Filter. *Sci. Rep.* **7**, 40550; doi: 10.1038/srep40550 (2017).

**Publisher's note:** Springer Nature remains neutral with regard to jurisdictional claims in published maps and institutional affiliations.

## Supplementary Material

Supplementary Information

## References

[b1] Liu C. . Transparent air filter for high-efficiency PM2.5 capture. Nat. Commun. 6, 6205 (2015). 25683688 10.1038/ncomms7205

[b2] Luo H. . Kaempferol nanoparticles achieve strong and selective inhibition of ovarian cancer cell viability. Int. J. Nanomed. 7, 3951–3959 (2012). 10.2147/IJN.S33670 PMC3410694 22866004

[b3] Lee E. S., Fung C. D. & Zhu Y. Evaluation of a high efficiency cabin air (HECA) filtration system for reducing particulate pollutants inside school buses. Environ. Sci. Technol. 49, 3358–3365 (2015). 25728749 10.1021/es505419m

[b4] Stephens B. & Siegel J. A. Ultrafine particle removal by residential heating, ventilating, and air-conditioning filters. Indoor Air 23, 488–497 (2013). 23590456 10.1111/ina.12045

[b5] Li M. . High performance filtration nanofibrous membranes based on hydrophilic poly(vinyl alcohol-co-ethylene) copolymer. Desalination 329, 50–56 (2013).

[b6] Uppal R., Bhat G., Eash C. & Akato K. Meltblown nanofiber media for enhanced quality factor. Fibers Polym. 14, 660–668 (2013).

[b7] Thomas D., Penicot P., Contal P., Leclerc D. & Vendel J. Clogging of fibrous filters by solid aerosol particles Experimental and modelling study. Chem. Eng. Sci. 56, 3549–3561 (2001).

[b8] Hung C. H. & Leung W. W. Filtration of nano-aerosol using nanofiber filter under low Peclet number and transitional flow regime. Sep. Purif. Technol. 79, 34–42 (2011).

[b9] Thakur R., Das D. & Das A. Electret Air Filters. Sep. Purif. Rev. 42, 87–129 (2012).

[b10] Barhate R. S. & Ramakrishna S. Nanofibrous filtering media: Filtration problems and solutions from tiny materials. J. Membr. Sci. 296, 1–8 (2007).

[b11] Barhate R. S., Loong C. K. & Ramakrishna S. Preparation and characterization of nanofibrous filtering media. J. Membr. Sci. 283, 209–218 (2006).

[b12] Gong J., Li X. D., Ding B., Lee D. R. & Kim H. Y. Preparation and characterization of H_4_SiMo_12_O_40_/poly(vinyl alcohol) fiber mats produced by an electrospinning method. J. Appl. Polym. Sci. 89, 1573–1578 (2003).

[b13] Si Y. . Superelastic and superhydrophobic nanofiber-assembled cellular aerogels for effective separation of oil/water emulsions. ACS Nano 9, 3791–3799 (2015). 25853279 10.1021/nn506633b

[b14] Lin J. . Facile control of intra-fiber porosity and inter-fiber voids in electrospun fibers for selective adsorption. Nanoscale 4, 5316–5320 (2012). 22837078 10.1039/c2nr31515g

[b15] Greiner A. & Wendorff J. H. Electrospinning: a fascinating method for the preparation of ultrathin fibers. Angew. Chem. Int. Ed. 46, 5670–5703 (2007). 10.1002/anie.200604646 17585397

[b16] Sambaer W., Zatloukal M. & Kimmer D. 3D modeling of filtration process via polyurethane nanofiber based nonwoven filters prepared by electrospinning process. Chem. Eng. Sci. 66, 613–623 (2011).

[b17] Zhang S., Liu H., Yin X., Yu J. & Ding B. Anti-deformed polyacrylonitrile/polysulfone composite membrane with binary structures for effective air filtration. ACS Appl. Mater. Interfaces 8, 8086–8095 (2016). 26958995 10.1021/acsami.6b00359

[b18] Yang Y., Zhang S., Zhao X., Yu J. & Ding B. Sandwich structured polyamide-6/polyacrylonitrile nanonets/bead-on-string composite membrane for effective air filtration. Sep. Purif. Technol. 152, 14–22 (2015).

[b19] Liu B., Zhang S., Wang X., Yu J. & Ding B. Efficient and reusable polyamide-56 nanofiber/nets membrane with bimodal structures for air filtration. J. Colloid Interface Sci. 457, 203–211 (2015). 26188726 10.1016/j.jcis.2015.07.019

[b20] Wang Z., Zhao C. & Pan Z. Porous bead-on-string poly(lactic acid) fibrous membranes for air filtration. J. Colloid Interface Sci. 441, 121–129 (2015). 25499733 10.1016/j.jcis.2014.11.041

[b21] Wang N., Wang X., Ding B., Yu J. & Sun G. Tunable fabrication of three-dimensional polyamide-66 nano-fiber/nets for high efficiency fine particulate filtration. J. Mater. Chem. 22, 1445 (2012).

[b22] Patanaik A., Jacobs V. & Anandjiwala R. D. Performance evaluation of electrospun nanofibrous membrane. J. Membr. Sci. 352, 136–142 (2010).

[b23] Przekop R. & Gradoń L. Effect of particle and fiber size on the morphology of deposits in fibrous filters. Int. J. Numer. Methods Fluids 76, 779–788 (2014).

[b24] Sahay R. . Electrospun composite nanofibers and their multifaceted applications. J. Mater. Chem. 22, 12953–12971 (2012).

[b25] Seo J. M., Arumugam G. K., Khan S. & Heiden P. A. Comparison of the effects of an ionic liquid and triethylbenzylammonium chloride on the properties of electrospun fibers, 1 – poly(lactic acid). Macromol. Mater. Eng. 294, 35–44 (2009).

[b26] Yarin A. L. Coaxial electrospinning and emulsion electrospinning of core–shell fibers. Polym. Adv. Technol. 22, 310–317 (2011).

[b27] Zhang Z. . Electrospun nanofibers of ZnO−SnO_2_ heterojunction with high photocatalytic activity. J. Phys. Chem. C 114, 7920–7925 (2010).

[b28] Chaobo H. . Electrospun polymer nanofibres with small diameters. Nanotechnology 17, 1558–1563 (2006). 26558558 10.1088/0957-4484/17/6/004

[b29] Ding B., Li C., Miyauchi Y., Kuwaki O. & Shiratori S. Formation of novel 2D polymer nanowebs via electrospinning. Nanotechnology 17, 3685–3691 (2006).

[b30] Zhang S., Chen K., Yu J. & Ding B. Model derivation and validation for 2D polymeric nanonets: Origin, evolution, and regulation. Polymer 74, 182–192 (2015).

[b31] Wang N. . Ultra-light 3D nanofibre-nets binary structured nylon 6–polyacrylonitrile membranes for efficient filtration of fine particulate matter. J. Mater. Chem. A 3, 23946–23954 (2015).

[b32] Zhang S., Liu H., Yu J., Luo W. & Ding B. Microwave structured polyamide-6 nanofiber/net membrane with embedded poly(m-phenylene isophthalamide) staple fibers for effective ultrafine particle filtration. J. Mater. Chem. A 4, 6149–6157 (2016).

[b33] Chen K. . Large-scale fabrication of highly aligned poly(m-phenylene isophthalamide) nanofibers with robust mechanical strength. RSC Advances 4, 45760–45767 (2014).

[b34] Yeom B. Y., Shim E. & Pourdeyhimi B. Boehmite nanoparticles incorporated electrospun nylon-6 nanofiber web for new electret filter media. Macromol. Res. 18, 884–890 (2010).

[b35] Liang T., Parhizkar M., Edirisinghe M. & Mahalingam S. Effect of humidity on the generation and control of the morphology of honeycomb-like polymeric structures by electrospinning. Eur. Polym. J. 61, 72–82 (2014).

[b36] Fashandi H. & Karimi M. Comparative studies on the solvent quality and atmosphere humidity for electrospinning of nanoporous polyetherimide fibers. Ind. Eng. Chem. Res. 53, 235–245 (2014).

[b37] Wang X. . Tuning hierarchically aligned structures for high-strength PMIA-MWCNT hybrid nanofibers. Nanoscale 5, 886–889 (2013). 23247563 10.1039/c2nr33696k

[b38] Yang S. . Controllable fabrication of soap-bubble-like structured polyacrylic acid nano-nets via electro-netting. Nanoscale 3, 564–568 (2011). 21060959 10.1039/c0nr00730g

[b39] Zaripov S. K., Soloveva O. V. & Skvortsov E. V. Analytical model of the transport of aerosol particles in a circular hole inside a porous medium. Transp. Porous Media 107, 141–151 (2015).

[b40] Sambaer W., Zatloukal M. & Kimmer D. 3D air filtration modeling for nanofiber based filters in the ultrafine particle size range. Chem. Eng. Sci. 82, 299–311 (2012).

[b41] Li L., Bellan L. M., Craighead H. G. & Frey M. W. Formation and properties of nylon-6 and nylon-6/montmorillonite composite nanofibers. Polymer 47, 6208–6217 (2006).

[b42] Wong S. C., Baji A. & Leng S. Effect of fiber diameter on tensile properties of electrospun poly(ɛ-caprolactone). Polymer 49, 4713–4722 (2008).

[b43] Papkov D. . Simultaneously strong and tough ultrafine continuous nanofibers. ACS nano 7, 3324–3331 (2013). 23464637 10.1021/nn400028p

[b44] Leung W. W., Hung C. H. & Yuen P. T. Effect of face velocity, nanofiber packing density and thickness on filtration performance of filters with nanofibers coated on a substrate. Sep. Purif. Technol. 71, 30–37 (2010).

[b45] Hollingsworth & Vose. Global manufacturer of advanced materials for filtration, battery, and industrial applications. http://www.hollingsworth-vose.com/en/Products/Filtration-Media/Air-Filtration.

[b46] Tronville P. & Rivers. R. Looking for the minimum efficiency of fibrous air filters during their service life 4, 1–8 (2012).

