# Supplementary material for: Tailoring Mechanically Robust Poly(m-phenylene isophthalamide) Nanofiber/nets for Ultrathin High-Efficiency Air Filter
Source: Sci Rep. 2017 Jan 11;7:40550. doi: 10.1038/srep40550 (PMC5225417; doi:10.1038/srep40550)
Supplement: Supplementary Information [file srep40550-s1.pdf]

Supplementary Information

Tailoring Mechanically Robust Poly(*m*-phenylene isophthalamide) Nanofiber/nets for Ultrathin High-Efficiency Air Filter

Shichao Zhang,[1,3] Hui Liu,[2,3] Xia Yin,[2] Zhaoling Li,[2] Jianyong Yu,[3] and Bin Ding*,[1,2,3]

[1] State Key Laboratory for Modification of Chemical Fibers and Polymer Materials, College of Materials Science and Engineering, Donghua University, Shanghai 201620, China.

[2] Key Laboratory of Textile Science & Technology, Ministry of Education, College of Textiles, Donghua University, Shanghai 201620, China.

[3] Nanofibers Research Center, Modern Textile Institute, Donghua University, Shanghai 200051, China.

[*] Corresponding author. E-mail: binding@dhu.edu.cn; Phone: +86-21-62378202; Fax: +86-21-62378202

Supporting Information contains:

Supplementary Theoretical Derivations

Supplementary Methods

Supplementary Discussions

Supplementary Scheme S1

Supplementary Figures S1-S6

Supplementary Tables S1-S6

Supplementary References

Derivation of the theoretical charge to mass ratio for ejecting charged droplets

Firstly, we can obtain the diameter D of the Taylor cone apex, namely the diameter of the charged liquid (jets and droplets) by inputting the solution properties into the following equation proposed in our previous work:

$$D = \frac{1.4639 Q^{0.44} \varepsilon^{0.12} \eta^{0.32}}{K^{0.12} \gamma^{0.32}}$$

Then, the critical condition of "droplet ejection" that both droplets and jets can be ejected from the Taylor cone apex is obtained, viz.,

$$\frac{e}{m} > D_c = \sqrt{\frac{288 \varepsilon \gamma}{\rho^2 D^3}} \cdot$$

The detailed results are demonstrated in Table S2.

Measurement of the experimental charge to mass ratio of the charged liquid

The typical method of mesh target was introduced to measure the charge to mass ratio of charged liquids. A high precision multi-meter (Fluke F15B+, Fluke electronic instrument Co., Ltd., China) connecting the high-voltage DC power supply and receiving substrate, was used to measure the current (I) induced by the moving residual charges on the liquids. During a certain spinning/netting time (t), the total charge (e) can be calculated by the formula of $e = I \times t$. Meanwhile, the mass of charged liquid (m) in this period can be calculated by the formula of $m = M/c$, where M represents the mass of membrane and c is the PMIA concentration. At least 5 parallel tests were carried out for each kind of sample for the above calculations. The detailed results are tabulated in Table S2.

Filtration measurement of the PMIA nanofibrous media

Here we used the LZC-H filter tester (Huada Filter Technology Co., Ltd.) to measure the filtration performance of the resultant membranes, as shown in Scheme S2. The fibrous membranes were

clamped by a filter holder with an effective area of 100 cm^2. As the 5.33 cm/s is usually regarded as the industrial standard flow rate, thus the air flow of 32 L/min can be calculated by employing the testing area of 100 cm^2. The QRJ-1 sodium chloride (NaCl) atomizer can generate NaCl aerosol particles with diameter of 0.3-10 μm at a flow rate of 20-35 L/min, and these NaCl particles are neutralized by capturing the ions with opposite charges generated by an electrostatic neutralization device. Then, 300,000-500,000 charge neutralized monodisperse solid NaCl aerosol particles are delivered through the testing filtration medium by the air pump. The particles that have a mass mean diameter of 300-500 nm and a geometric standard deviation not exceeding 1.86, are feed into the filter holder and down through the filter at a continuous airflow speed of 32 L/min. The filtration efficiency is measured via detecting the number of NaCl particles in the upstream and downstream of the particles airflow by two BCJ-1K laser particle counters, respectively. While, the filter resistance is obtained via the combination of a flow meter and two electronic pressure transducers that can detect the pressure drop through the filtration medium under testing. For measuring the dust-holding capacity of the filters, the testing particles are substituted by the silica nanoparticles in the size range of 4-70 nm which are uniformly placed in a metal circular groove to shorten the testing time. The dust-holding capacity can be calculated by measuring the weight change of the media between the initial state and end-of-life of 1000 Pa. All tests were conducted at the room temperature of 25 ^oC and the relative humidity of 50%.

Additionally, the long-term purification test towards smoke $PM_{2.5}$ by the PMIA NF/N filter of 99.999% removal efficiency for NaCl particles was also carried out in an enclosed cabin of 0.1 m^3. The model PM were generated by burning the cigarettes and showed broad diameter distribution from <0.3 μm to >10 μm, and most of them were in the range of <1 μm. The severely polluted air ($PM_{2.5}$ concentration >500 $\mu g/m^3$) was fed through the PMIA NF/N filter with area of ~24 cm^2 under a

continue airflow of 14 L/min using a simple fan. Then we can test the real-time value of $PM_{2.5}$ concentration in the cabin by the detection instrument (SDL 301, Nova Fitness). We recorded the time it required until $PM_{2.5}$ concentration came down to 35 μg/m^3. And, the long-term recycling operational performance was evaluated by testing 30 cycles.

Mechanical properties of the PMIA nanofibers

From the Figure 1g we can know that, the tensile stress of PMIA nanofiber membranes fabricated from 10, 12, 14, and 16 wt% PMIA solutions were 26.17, 35.30, 42.93, and 52.26 MPa, respectively, indicating a regular increase of tensile strength upon increasing the solution concentration. Further analysis for the stress-strain curves presented that, with increasing the PMIA concentrations, the PMIA membranes possessed a toughness of 3.44, 5.57, 7.86, and 11.04 MJ/m^3,and a Young's modulus of 0.2876, 0.3117, 0.3242, and 0.3272 GPa, respectively, as demonstrated in Figure S1. This significant enhancement of the mechanical properties was mainly due to the transition of nanofibers structures including increased fiber diameter and uniform fiber diameter distribution, which was well consistent with the results in our previous report about the aligned PMIA fibers with robust mechanical strength.[1,2]

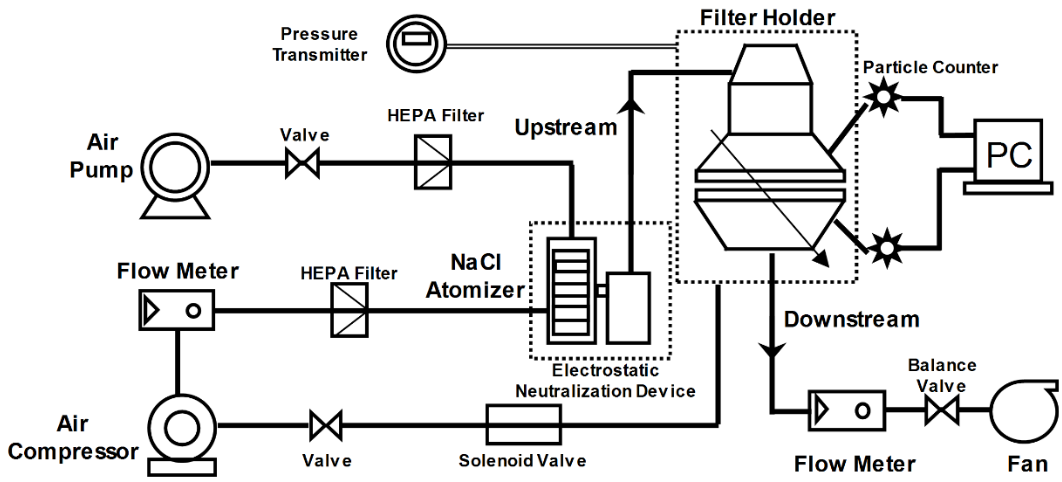

Scheme S1. Schematic diagram of the experimental setup for evaluating the filtration performance of the filter media.

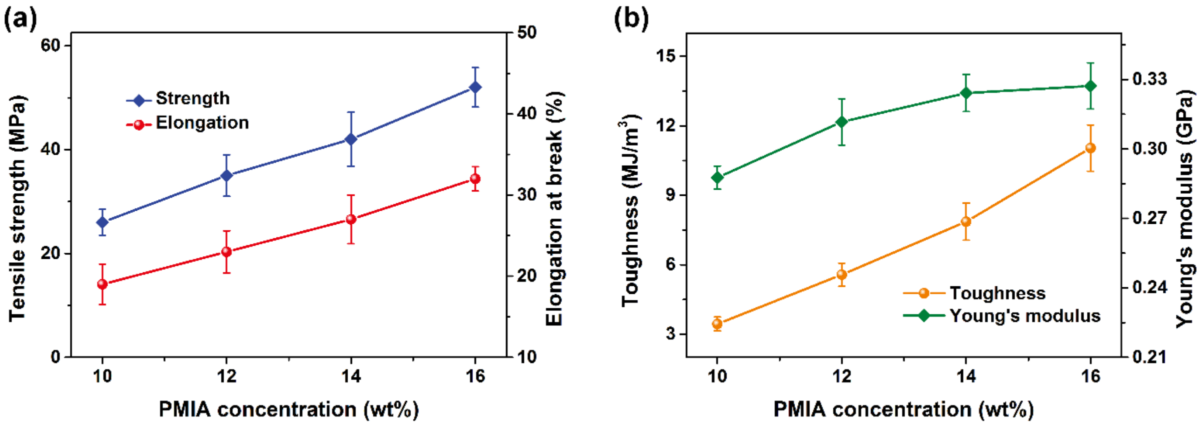

Figure S1. (a) Tensile strength and elongation at break, (b) toughness and Young's modulus of PMIA nanofiber membranes fabricated from PMIA solutions with various concentrations at RH of 40%.

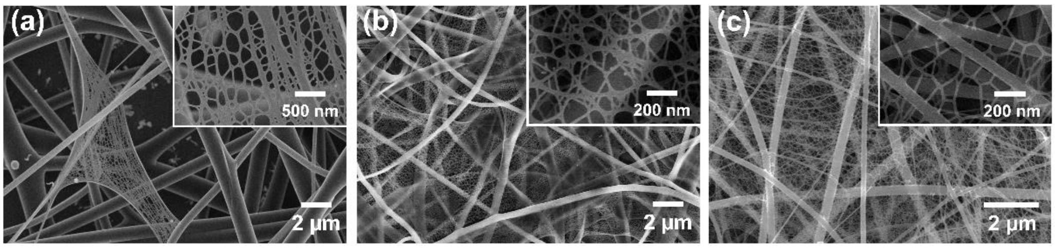

Figure S2. Representative FE-SEM images of (a) PAA, (b) PA-6, and (c) PMIA nanofiber/nets membranes. The insets are the magnified images of the corresponding nanonets structures.

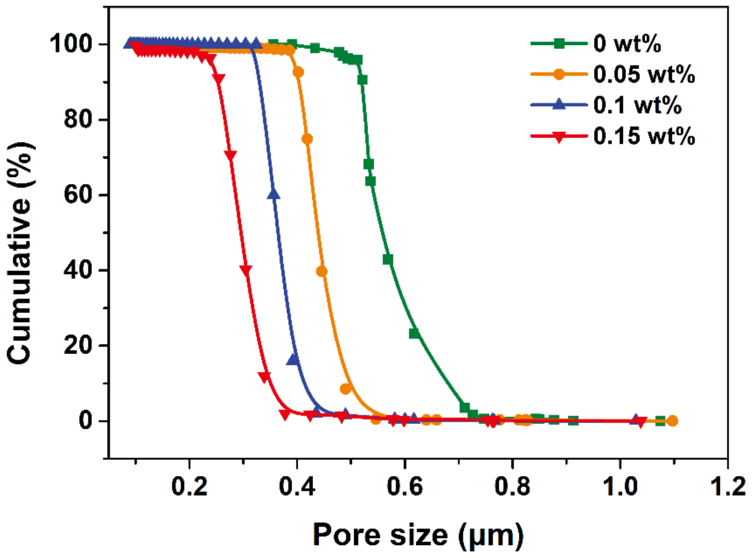

Figure S3. Cumulative porosity distribution curves of PMIA NF/N membranes (0.125 g/m^2) with different DTAB concentrations.

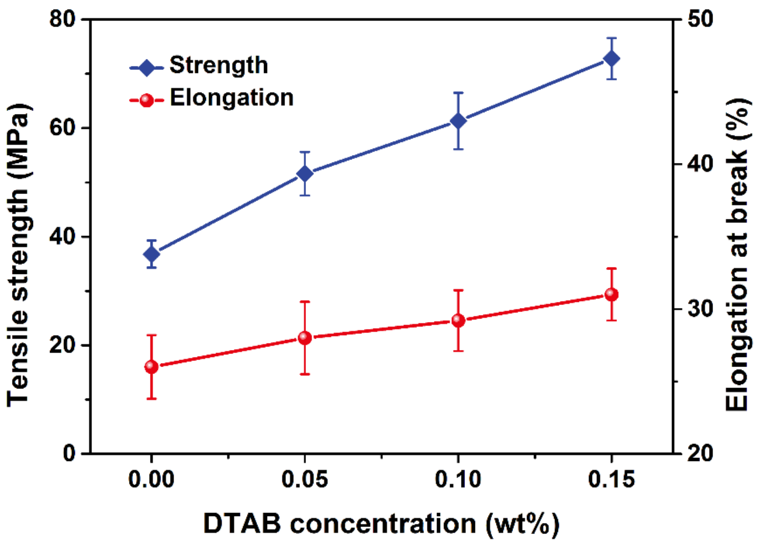

Figure S4. Tensile strength and elongation at break of PMIA NF/N membranes prepared from PMIA solutions with different DTAB concentrations at RH of 25%.

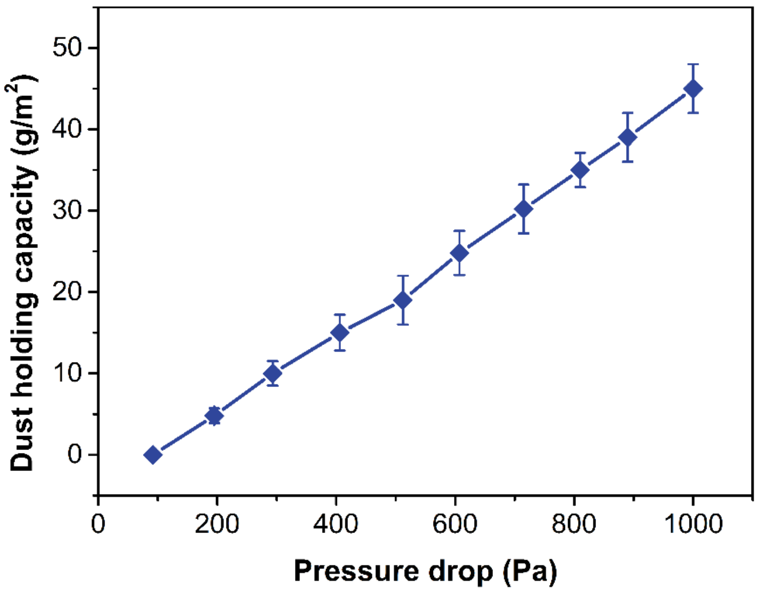

Figure S5. Dust holding capacity of PMIA NF/N filter at different pressure drops during a continuous silica particles feed.

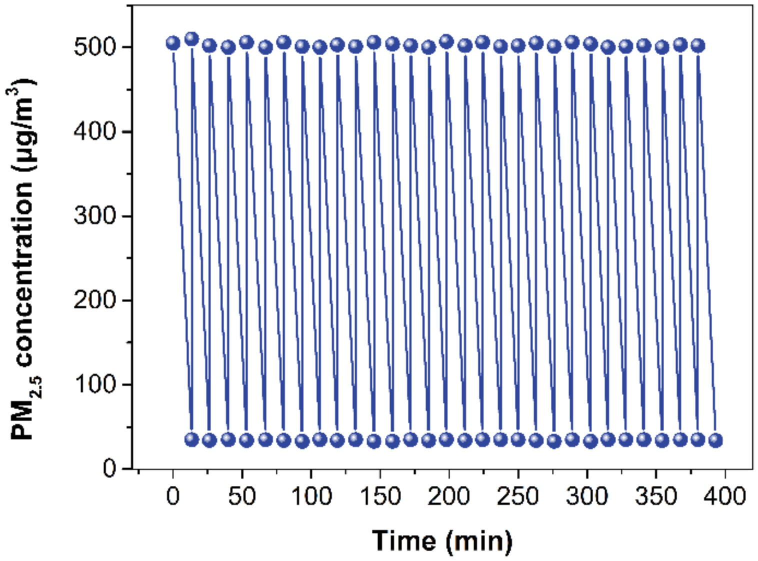

Figure S6. Long-term recycling performance of PMIA NF/N filter for removing smoke $PM_{2.5}$ from >500 µg/m^3 to <35 µg/m^3.

Table S1. Composition and properties of various electrospinning/netting solutions.

**PMIA (wt%)**	**LiCl (wt%)**	**DTAB (wt%)**	**Viscosity (cps)**	**Conductivity (mS cm^{-1})**	**Surface tension (mN m^{-1})**
10	2.5	0	1257	3.42	37.60
12	2.5	0	3920	3.15	37.40
14	2.5	0	4800	2.68	37.20
16	2.5	0	10307	2.12	37.01
12	2.5	0.05	3830	3.16	34.82
12	2.5	0.1	3750	3.19	33.04
12	2.5	0.15	3700	3.21	31.89
12	2.5	0.2	3650	3.26	30.02

Table S2. Effect of DTAB concentration and RH on the charge to mass ratio of the fluid during ESN

	**RH**	**25%**	**40%**	**25%**	**25%**
**Theoretical analysis**	**DTAB concentration (wt %)**	**0**	**0**	**0.05**	**0.15**
	ε (F/m)	24.5*8.85*10^-12			
	γ (mN/m)	37.4		34.82	31.89
	ρ (kg/m^3)	1016.7		1016.9	1017.2
	η (cps)	3920		3830	3700
	K (mS/cm)	3.15		3.16	3.21
	Q (mL/h)	0.1			
	D (m)	1.169*10^-6		1.187*10^-5	1.205*10^-5
	$\sqrt{\frac{64\varepsilon\gamma}{\rho^2 D^3}}$ (c/kg)	0.5606		0.5288	0.4945
	$\sqrt{\frac{288\varepsilon\gamma}{\rho^2 D^3}}$ (c/kg)	1.189		1.122	1.049
**Experimental analysis**	$\frac{e}{M}$ (c/kg)	1.325	0.0774	2.156	3.968

Table S3. Pore size and porosity of PMIA NF/N membranes with various DTAB concentrations.

Samples	DTAB (wt%)	Maximum pore size (μm)	Mean flow pore size (μm)	Porosity (%)
PMIA NF/N membranes	0	0.8545	0.5642	65.13
	0.05	0.7272	0.4377	72.39
	0.1	0.5490	0.3654	77.50
	0.15	0.3782	0.2960	80.25

Table S4. BET surface area of PMIA NF/N membranes with various DTAB concentrations.

**Samples**	**DTAB contents (wt%)**	**BET surface area (m^2/g)**
PMIA NFN membranes	0	17.5513
	0.05	21.2768
	0.1	24.6595
	0.15	26.8870

Table S5. Comparison of filtration efficiency, pressure drop, and basis weight between current filtration materials and PMIA NF/N membranes.

Materials	Polymers/grade		Basis weight (g/m^2)	Filtration efficiency (%)	Pressure drop (Pa)	Ref.
**Electrospun nanofibers**	PA6		10.75	99.993	1470	[3]
	PAN		3.895	99.999	356	[4]
	PLA		5.21	99.997	165.3	[5]
	PVC/PU		20.72	99.5	144	[6]
	PAN/PU		24.04	99.98	120	[7]
	PAN/SiO_2		2.04	99.989	117	[8]
	PSU/TiO_2		7.75	99.997	45.3	[9]
	PEI/SiO_2		5.64	99.992	61	[10]
**H&V glass medias**	Standard Glass Grades US	HA 9653	78	99.999996	677	[11]
		HA 8603	78	99.9991	372	
		HB 7613	78	99.996	343	
		HYB5639	68	99.985	294	
	Standard Glass Media EMEA	HA8303	78	99.99985	495	
		HA8323	78	99.99955	435	
		HB5773	78	99.9994	405	
		HB5451	90	99.995	352	
		HB5703	78	99.944	305	
		HC4313	76	99.85	285	
		HC3313	75	99.6	245	
	High Strength Glass Media EMEA	HC15826	81	99.941	280	
**H&V meltblown fibers**	Technostat - Pleated Filters	T300(+)15	315	99.992	22	
		T250(+)15	265	99.963	14.5	

	T200(+)15	215	99.9	12
	T150(+)15	165	99.8	9.17
	T100(+)15	115	99	6.17
	T70(+)15	85	95	4.17

Table S6. Comparison of tensile strength between current electrospun nanofiber filters and PMIA NF/N filter.

**Materials**	**PMIA NF/N**	**Electrospun nanofibers**						
**Polymer**	PMIA	Silk	PVC/PU	PAN/PU	PEI/SiO_2	PA-56	PAN/PSU	PA-6
**Tensile strength (MPa)**	72.8	42	9.9	12.28	5.79	11.02	8.2	3.65

References

[1] K. Chen, S. Zhang, B. Liu, X. Mao, G. Sun, J. Yu, S.S. Al-Deyab, B. Ding, Large-scale fabrication of highly aligned poly(m-phenylene isophthalamide) nanofibers with robust mechanical strength, RSC Adv., 4 (2014) 45760-45767.

[2] X. Wang, Y. Si, J. Yang, B. Ding, L. Chen, Z. Hu, J. Yu, Tuning hierarchically aligned structures for high-strength PMIA-MWCNT hybrid nanofibers, Nanoscale, 5 (2013) 886-889.

[3] G. Kim, Y. Ahn, J. Lee, Characteristics of Nylon 6 nanofilter for removing ultra fine particles, Korean J. Chem. Eng., 25 (2008) 368-372.

[4] Y. Mei, Z. Wang, X. Li, Improving filtration performance of electrospun nanofiber mats by a bimodal method, J. Appl. Polym. Sci., 128 (2013) 1089-1094.

[5] Z. Wang, C. Zhao, Z. Pan, Porous bead-on-string poly(lactic acid) fibrous membranes for air filtration, J. Colloid Interf. Sci., 441 (2015) 121-129.

[6] N. Wang, A. Raza, Y. Si, J. Yu, G. Sun, B. Ding, Tortuously structured polyvinyl chloride/polyurethane fibrous membranes for high-efficiency fine particulate filtration, J. Colloid Interf. Sci., 398 (2013) 240-246.

[7] N. Wang, Z. Zhu, J. Sheng, S.S. Al-Deyab, J. Yu, B. Ding, Superamphiphobic nanofibrous membranes for effective filtration of fine particles, J. Colloid Interf. Sci., 428 (2014) 41-48.

[8] N. Wang, Y. Si, N. Wang, G. Sun, M. El-Newehy, S.S. Al-Deyab, B. Ding, Multilevel structured polyacrylonitrile/silica nanofibrous membranes for high-performance air filtration, Sep. Purif. Technol., 126 (2014) 44-51.

[9] H. Wan, N. Wang, J. Yang, Y. Si, K. Chen, B. Ding, G. Sun, M. El-Newehy, S.S. Al-Deyab, J. Yu, Hierarchically structured polysulfone/titania fibrous membranes with enhanced air filtration performance, J. Colloid Interf. Sci., 417 (2014) 18-26.

[10] X. Li, N. Wang, G. Fan, J. Yu, J. Gao, G. Sun, B. Ding, Electreted polyetherimide–silica fibrous membranes for enhanced filtration of fine particles, J. Colloid Interf. Sci., 439 (2015) 12-20.

[11] Hollingsworth & Vose, Global manufacturer of advanced materials for filtration, battery, and industrial applications, in, http://www.hollingsworth-vose.com/en/Products/Filtration-Media/Air-Filtration1/
